# Supplementary material for: Smooth Interpolating Curves with Local Control and Monotone Alternating Curvature
Source: Comput Graph Forum. 2022 Oct 6;41(5):25–38. doi: 10.1111/cgf.14600 (PMC9827861; doi:10.1111/cgf.14600)
Supplement: Supplementary file 1 — Supplement Material [file CGF-41-25-s001.zip › Local-Smooth-Interpolating-MonoCurvature/extern/clothoids/submodules/Utils/docs/api-cpp/program_listing_file_Numbers.cc.html]

Program Listing for File Numbers.cc — Utils v1.2

### Navigation

- index
- toc
- Utils »
- Program Listing for File Numbers.cc

# Program Listing for File Numbers.cc¶

↰ Return to documentation for file (`Numbers.cc`)

```
/*--------------------------------------------------------------------------*\
 |                                                                          |
 |  Copyright (C) 2020                                                      |
 |                                                                          |
 |         , __                 , __                                        |
 |        /|/  \               /|/  \                                       |
 |         | __/ _   ,_         | __/ _   ,_                                |
 |         |   \|/  /  |  |   | |   \|/  /  |  |   |                        |
 |         |(__/|__/   |_/ \_/|/|(__/|__/   |_/ \_/|/                       |
 |                           /|                   /|                        |
 |                           \|                   \|                        |
 |                                                                          |
 |      Enrico Bertolazzi                                                   |
 |      Dipartimento di Ingegneria Industriale                              |
 |      Universita` degli Studi di Trento                                   |
 |      email: enrico.bertolazzi@unitn.it                                   |
 |                                                                          |
\*--------------------------------------------------------------------------*/

#ifndef DOXYGEN_SHOULD_SKIP_THIS

#include "Utils.hh"

#ifdef __clang__
#pragma clang diagnostic ignored "-Wpoison-system-directories"
#endif

namespace Utils {

  //============================================================================
  /*    __                       _ _   _       _   _
  //   / _| ___  _   _ _ __   __| | \ | | __ _| \ | |
  //  | |_ / _ \| | | | '_ \ / _` |  \| |/ _` |  \| |
  //  |  _| (_) | |_| | | | | (_| | |\  | (_| | |\  |
  //  |_|  \___/ \__,_|_| |_|\__,_|_| \_|\__,_|_| \_|
  */
  bool
  foundNaN( double const * const pv, int DIM ) {
    for ( int i = 0; i < DIM; ++i )
      if ( !isRegular(pv[i]) )
        return true;
    return false;
  }

  bool
  foundNaN( float const * const pv, int DIM ) {
    for ( int i = 0; i < DIM; ++i )
      if ( !isRegular(pv[i]) )
        return true;
    return false;
  }

  /*       _               _    _   _       _   _
  //   ___| |__   ___  ___| | _| \ | | __ _| \ | |
  //  / __| '_ \ / _ \/ __| |/ /  \| |/ _` |  \| |
  // | (__| | | |  __/ (__|   <| |\  | (_| | |\  |
  //  \___|_| |_|\___|\___|_|\_\_| \_|\__,_|_| \_|
  */

  #define LINE_LINE_LINE_LINE "--------------------------------------------------------------------------------"

  void
  checkNaN(
    double const * const pv,
    char   const * const v_name,
    int                  DIM,
    int                  line,
    char   const * const file
  ) {
    for ( int i = 0; i < DIM; ++i ) {
      if ( isInfinite(pv[i]) ) {
        UTILS_ERROR(
          "{}\n({}):{}) found Infinity at {}[{}]\n{}\n",
          LINE_LINE_LINE_LINE,
          basename(file), line, v_name, i,
          LINE_LINE_LINE_LINE
        );
      } else if ( isNaN(pv[i]) ) {
        UTILS_ERROR(
          "{}\n({}):{}) found NaN at {}[{}]\n{}\n",
          LINE_LINE_LINE_LINE,
          basename(file), line, v_name, i,
          LINE_LINE_LINE_LINE
        );
      }
    }
  }

  void
  checkNaN(
    float const * const pv,
    char  const * const v_name,
    int                 DIM,
    int                 line,
    char  const * const file
  ) {
    for ( int i = 0; i < DIM; ++i ) {
      if ( isInfinite(pv[i]) ) {
        UTILS_ERROR(
          "{}\n({}):{}) found Infinity at {}[{}]\n{}\n",
          LINE_LINE_LINE_LINE,
          basename(file), line, v_name, i,
          LINE_LINE_LINE_LINE
        );
      } else if ( isNaN(pv[i]) ) {
        UTILS_ERROR(
          "{}\n({}):{}) found NaN at {}[{}]\n{}\n",
          LINE_LINE_LINE_LINE,
          basename(file), line, v_name, i,
          LINE_LINE_LINE_LINE
        );
      }
    }
  }
}

#endif
```

### Quick search

### Table of Contents

- rang - Colors for your Terminal
- Zstr
- C++ API

«
hide menu

menu
sidebar
»

### Navigation

- index
- toc
- Utils »
- Program Listing for File Numbers.cc

© Copyright 2021, Enrico Bertolazzi.
Created using Sphinx 4.2.0.
